# INEQUALITIES IN HEALTHY AGING: THE DIFFERENTIAL IMPACT OF EDUCATION AND WEALTH ACROSS COHORT STUDIES

**DOI:** 10.1093/geroni/igz038.2942

**Published:** 2019-11-08

**Authors:** Yu-Tzu Wu, Christina Daskalopoulou, Graciela Muniz Terrera, Martin Prince, Matthew Prina

**Affiliations:** 1 Social Epidemiology Research Group, Health Service and Population Research Department, Institute of Psychiatry, Psychology & Neuroscience, King’s College London, London, England, United Kingdom; 2 Centre For Clinical Brain Sciences, University Of Edinburgh, Edinburgh, Scotland, United Kingdom; 3 Global Health Institute, King’s College London, London, England, United Kingdom

## Abstract

Several studies have investigated longitudinal changes in health status and functional ability but few have examined whether inequalities in healthy ageing varied across different countries. The aim of this study is to investigate trajectories of health metric scores (generated in previous symposium abstract) over the ageing process and examine the impact of education and wealth on the trajectories across eight cohorts in the ATHLOS consortium (N=135,828) using multilevel regression modelling. After adjusting for age, gender and study, higher levels of education (9.52; 95% CI: 9.30, 9.74) and wealth (8.06; 95% CI: 7.84, 8.28) were associated with higher baseline scores but had minimal impacts on decline rates. These effect sizes varied across different cohort studies and the inequality gradient was found to be strongest in the Health Retirement Study from US. Future research may investigate potential mechanisms which might explain the differential impact of education and wealth in different societies.

